# Supplementary material for: KRAS interaction with RAF1 RAS-binding domain and cysteine-rich domain provides insights into RAS-mediated RAF activation
Source: Nat Commun. 2021 Feb 19;12:1176. doi: 10.1038/s41467-021-21422-x (PMC7895934; doi:10.1038/s41467-021-21422-x)
Supplement: Supplementary file 1 — Supplementary Information [file 41467_2021_21422_MOESM1_ESM.pdf]

KRAS interaction with the RAF1 RAS-binding domain and the cysteine-rich domain provides insights into RAS-mediated RAF activation

Timothy H. Tran[1,§], Albert H. Chan[1,§], Lucy C. Young[2,§], Lakshman Bindu[1], Chris Neale[3], Simon Messing[1], Srisathiyanarayanan Dharmaiah[1], Troy Taylor[1], John-Paul Denson[1], Dominic Esposito[1], Dwight V. Nissley[1], Andrew G. Stephen[1], Frank McCormick[1,2,#], Dhirendra K. Simanshu[1,#]

[1]NCI RAS Initiative, Cancer Research Technology Program, Frederick National Laboratory for Cancer Research, Leidos Biomedical Research, Inc., Frederick, MD 21702. [2]Helen Diller Family Comprehensive Cancer Center, University of California, San Francisco, CA 94158. [3]Theoretical Biology and Biophysics, Los Alamos National Laboratory, Los Alamos, NM 87545.

Supplementary Information

- Supplementary Figures 1-9
- Supplementary Tables 1-5
- Supplementary References

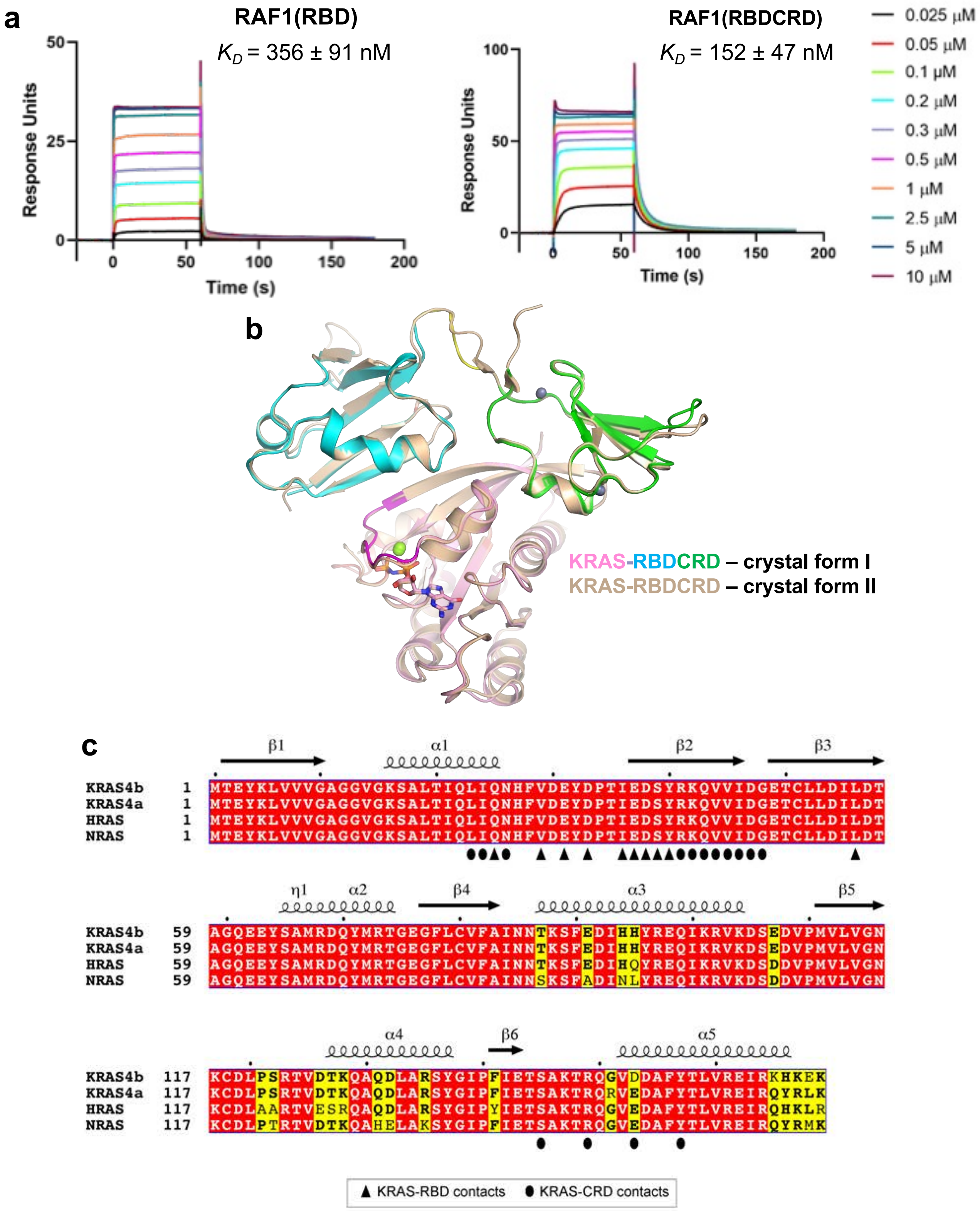

Supplementary Figure 1: SPR binding kinetics, comparison of two crystal forms of KRAS-RBDCRD complex, and conservation of RBDCRD interacting residues in RAS isoforms. (a) SPR sensorgrams showing the binding of RAF1(RBD) and RAF1(RBDCRD) to KRAS. RBD and RBDCRD (0.025 – 10 μM) were injected over avi-tagged WT KRAS, and binding responses were determined. The K_D values are the mean with the standard deviation from multiple replicates; RBD (n=18) and RBDCRD (n=17). Source data are provided as a Source Data file. **(b)** Structural superposition of the KRAS-RBDCRD complex solved in two different crystal forms shows a similar

arrangement of KRAS and RBDCRD inside the crystal. Crystal form I is colored using the same color scheme as Figure 1c, whereas crystal form II is colored in light brown color. **(c)** Amino acid sequence alignment of the G-domain of human KRAS4b, KRAS4a, HRAS, and NRAS. Fully and partially conserved residues among the RAS isoforms are highlighted in red and yellow, respectively. The secondary structural elements of KRAS seen in the KRAS-RBDCRD structure are shown above the alignment. The KRAS residues that are involved in the interaction with RBD and CRD are indicated below the alignment with upright triangles, and ovals, respectively.

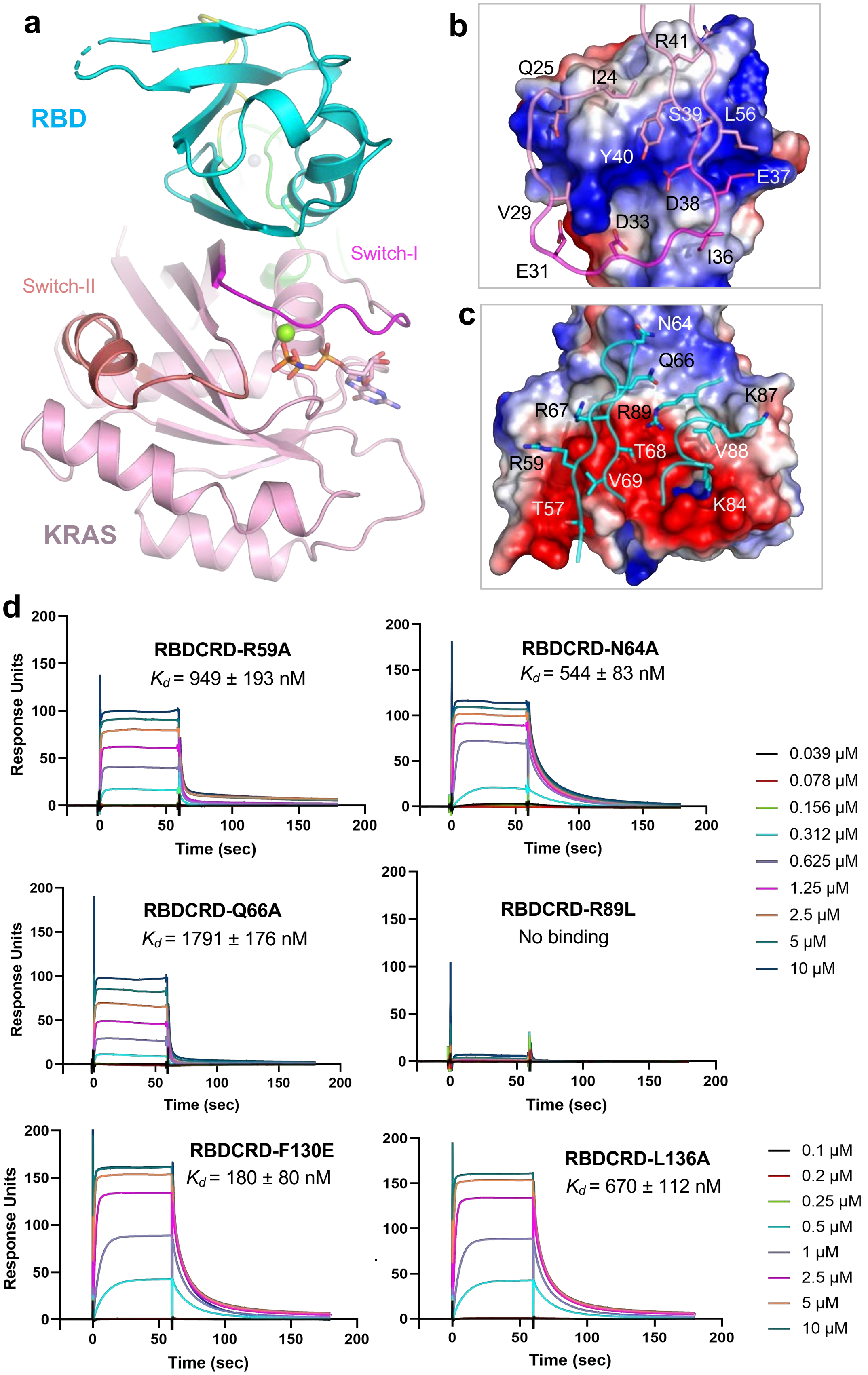

Supplementary Figure 2: Structural and mutational analysis of KRAS and RBD residues present at the interface in the KRAS-RBDCRD complex. (a) The overall structure of the KRAS-RBDCRD complex shown in cartoon representation, highlighting the KRAS-RBD interface. **(b)** The KRAS-RBD interaction interface in the KRAS-RBDCRD structure. RBD is shown in electrostatic

surface representation and the KRAS residues that participate at the interface are shown in stick representation. **(c)** The KRAS-RBD interaction interface in the KRAS-RBDCRD structure. KRAS is shown in electrostatic surface representation and the RBD residues that participate at the interface are shown in stick representation. **(d)** SPR sensorgrams showing the binding of RAF1(RBDCRD) mutants to WT KRAS. RBDCRD mutants R59A, N64A, Q66A, and R89L, were injected over avi-tagged WT KRAS in the concentration range (0.039 – 10 μM), and binding responses were determined. RBDCRD mutant F130E and L136A were injected over avi-tagged WT KRAS in the concentration range 0.1 - 20 μM. The K_D values are the mean from multiple replicates with the standard deviation. Source data are provided as a Source Data file.

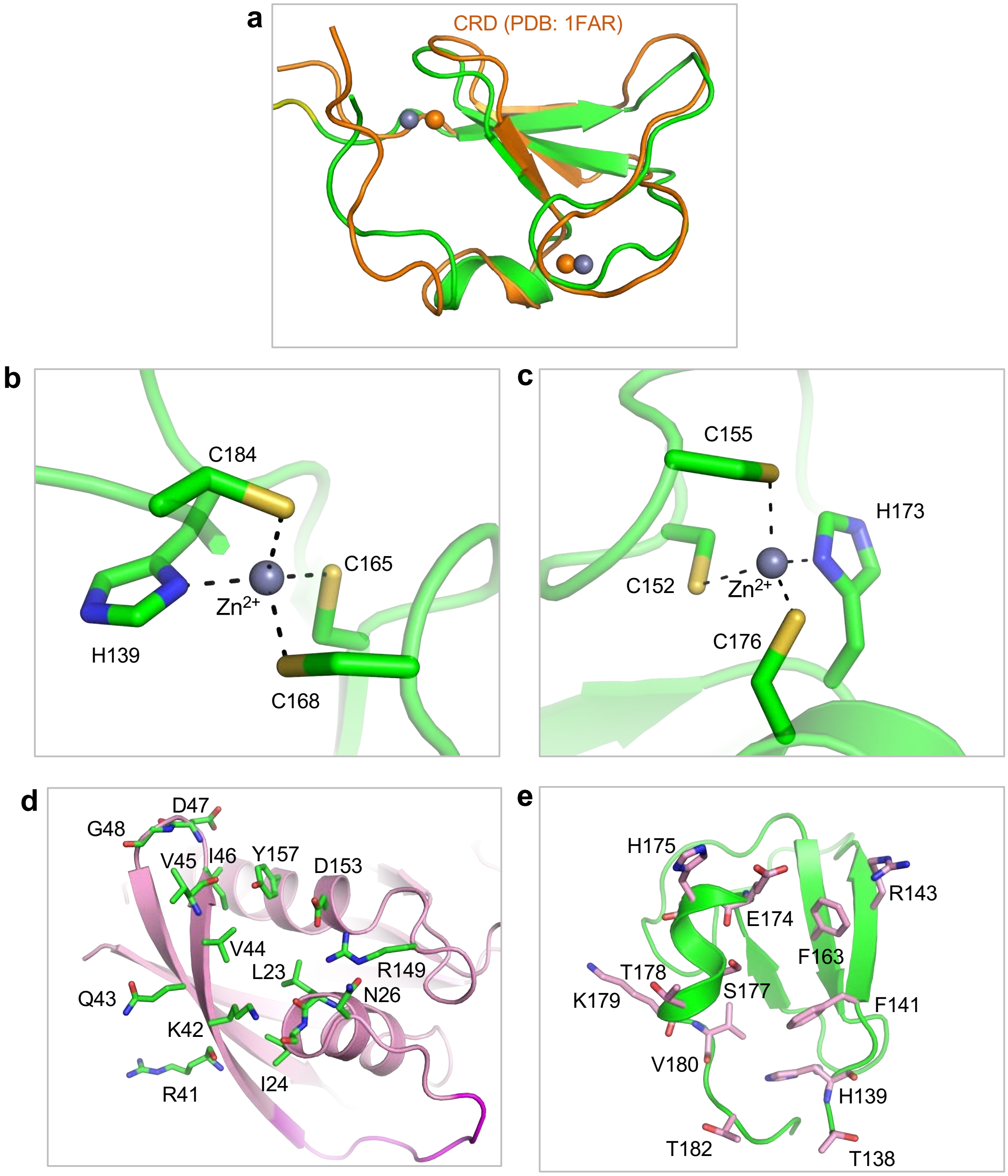

Supplementary Figure 3: Comparison of NMR and crystal structures of CRD, structural analysis of zinc fingers, and residues present at the KRAS-CRD interface. (a) Structural superposition of CRD observed in the KRAS-RBDCRD complex with the NMR (minimized average) structure of CRD (PDB ID: 1FAR) shows overall structural similarity. (**b, c**) Two zinc-finger motifs present in the CRD region of RBDCRD. Zinc(II) ions are shown as spheres, whereas CRD residues are shown in stick representation. Metal coordination bonds are indicated by dashed black lines. **(d)** KRAS (colored pink) is shown in cartoon representation with residues interacting with CRD highlighted in green. Main chains are shown for those residues that partly or solely interact with CRD via their main chain atoms. **(e)** CRD (colored green) is shown in cartoon representation with residues interacting with KRAS highlighted in pink. Main chains are shown for those residues that partly or solely interact with KRAS via their main chain atoms.

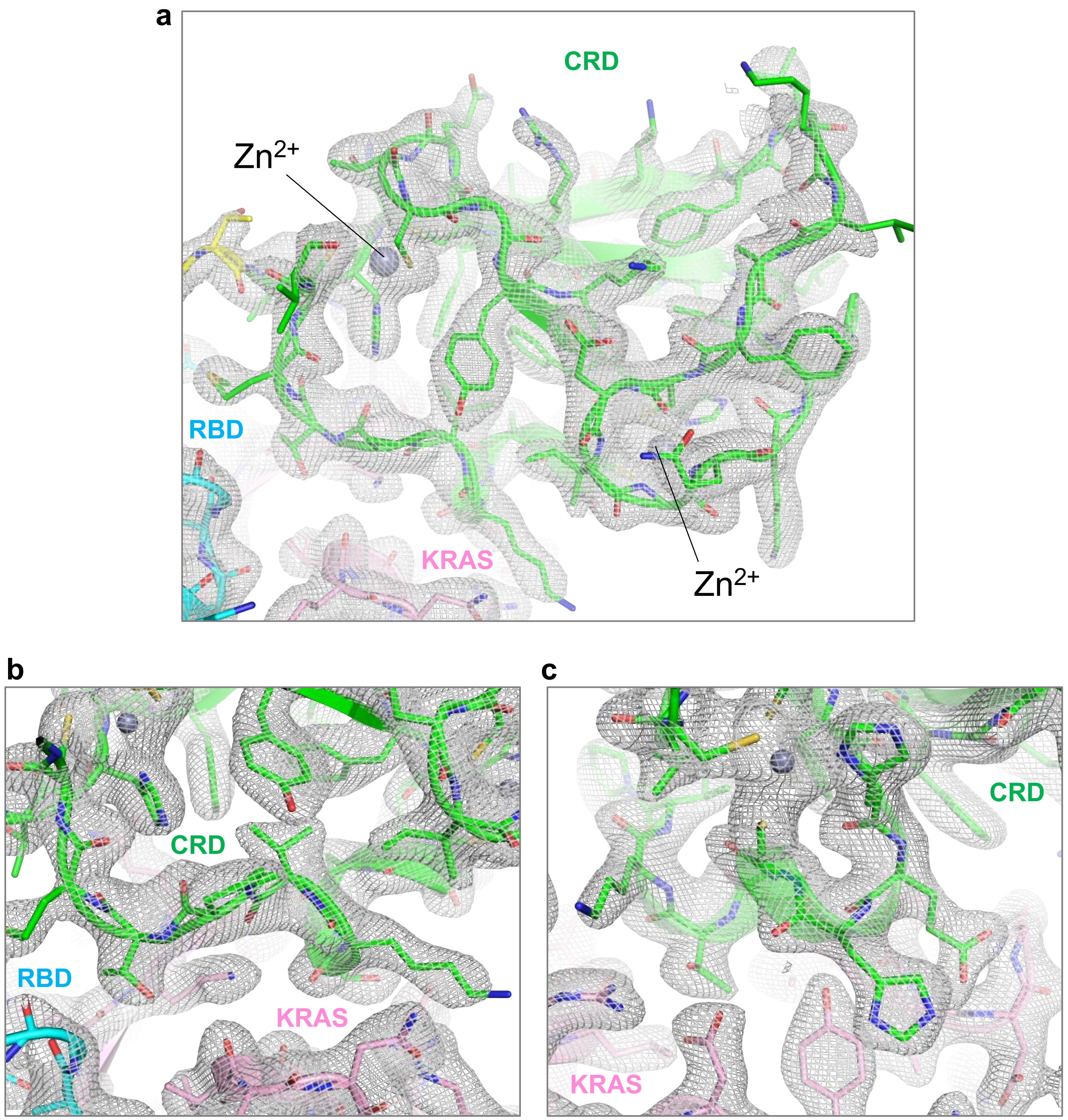

Supplementary Figure 4: Electron density map for the CRD and KRAS-CRD interface. **(a)** The *2Fo-Fc* map (contoured at 1 σ) corresponding for the RAF1(CRD) observed in the crystal form I **(b, c)** The *2Fo-Fc* map (contoured at 1 σ) corresponding for the KRAS-CRD interfaces shown in Fig. 4d and 4e.

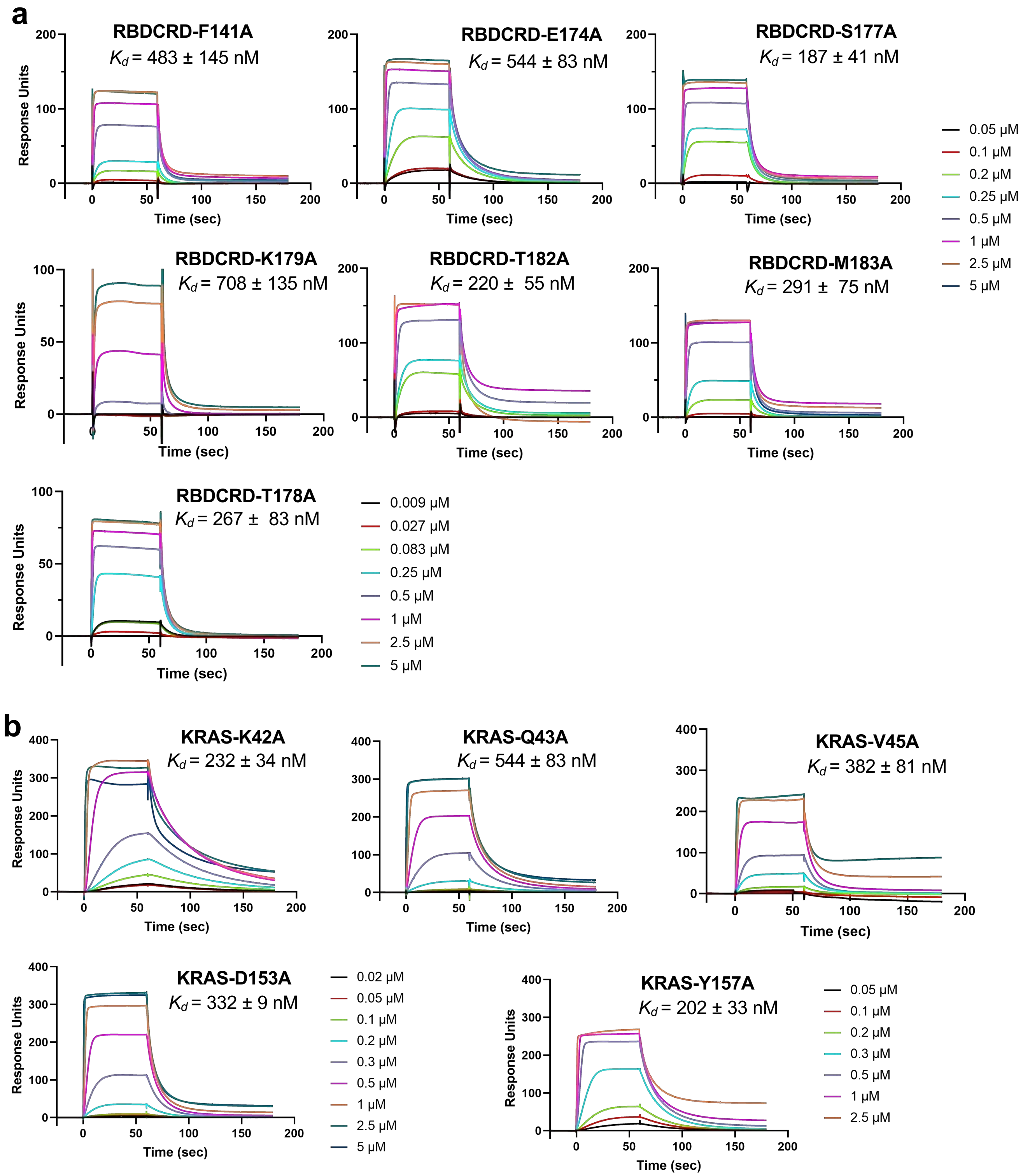

Supplementary Figure 5: SPR binding analysis of RBDCRD and KRAS mutants present at the KRAS-CRD interface. (a) SPR sensorgrams showing the binding of RAF1(RBDCRD) mutants to WT KRAS. RBDCRD mutants F141A, E174A, S177A, T178A, K179A, T182A, and M183A, were injected over avi-tagged WT KRAS in the concentration range (0.009 – 5 μM), and binding responses were determined. **(b)** SPR sensorgrams showing the binding of KRAS mutants to WT RBDCRD. WT RBDCRD was injected over avi-tagged KRAS mutant in the concentration range (0.02 - 5 μM), and binding responses were determined. For the KRAS mutant Y157A, WT RBDCRD was injected over avi-tagged KRAS-Y157A in the concentration range 0.05 - 2.5 μM. The K_D values are the mean from multiple replicates with the standard deviation. Source data are provided as a Source Data file.

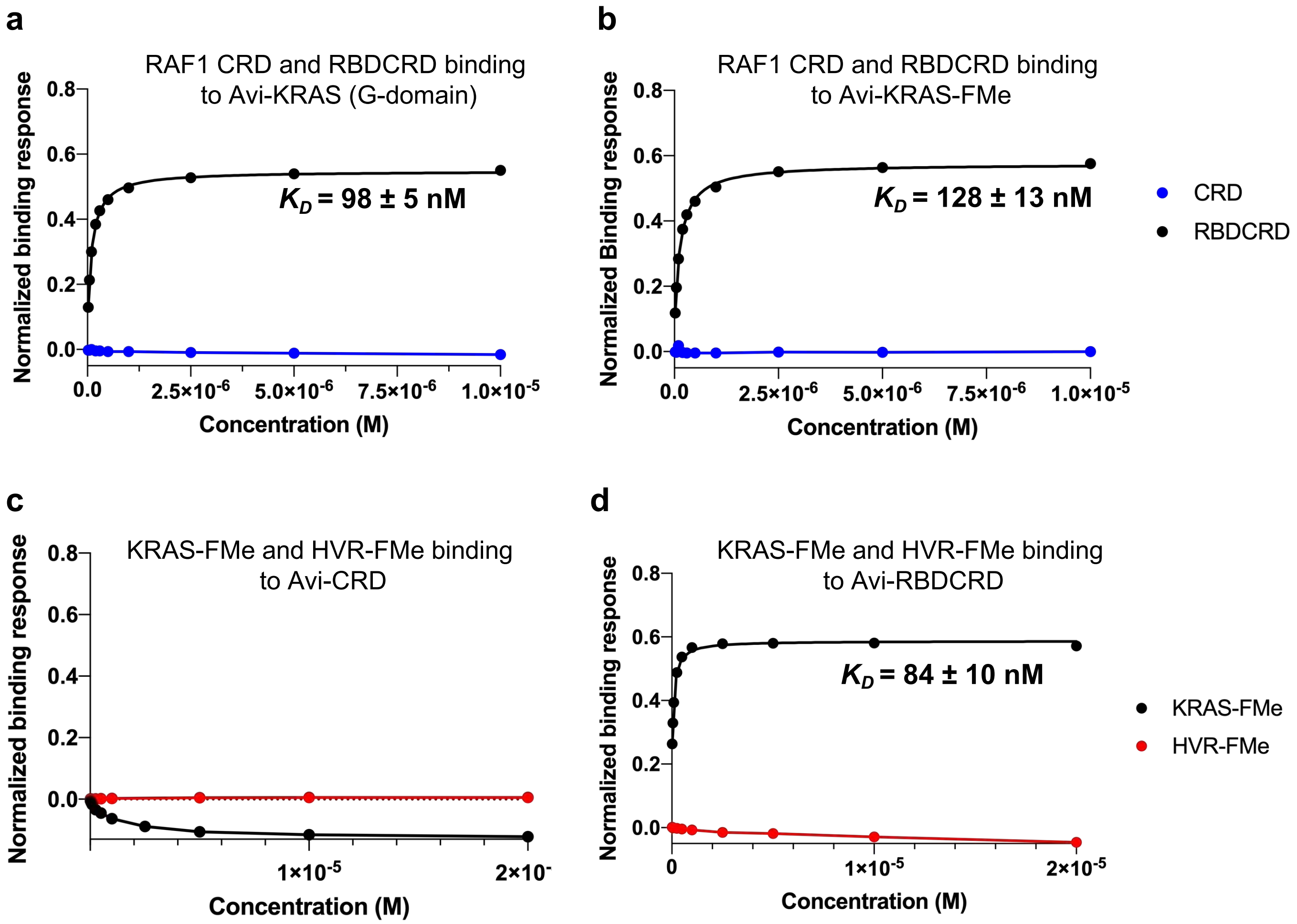

Supplementary Figure 6: SPR binding analysis to examine the role of the farnesylated hypervariable region of KRAS in KRAS-RAF1(CRD) interaction. (a, b) Steady-state binding isotherms derived from the SPR data of RAF1(CRD) and RAF1(RBDCRD) binding to (a) non-processed KRAS G-domain and (b) fully processed KRAS (KRAS-FMe). (**c, d**) Steady-state binding isotherms derived from the SPR data of KRAS-FMe and HVR-FMe peptide binding to (c) RAF1(CRD) and (d) RAF1(RBDCRD). The binding affinity is reported as K_D ± the standard error of the fit.

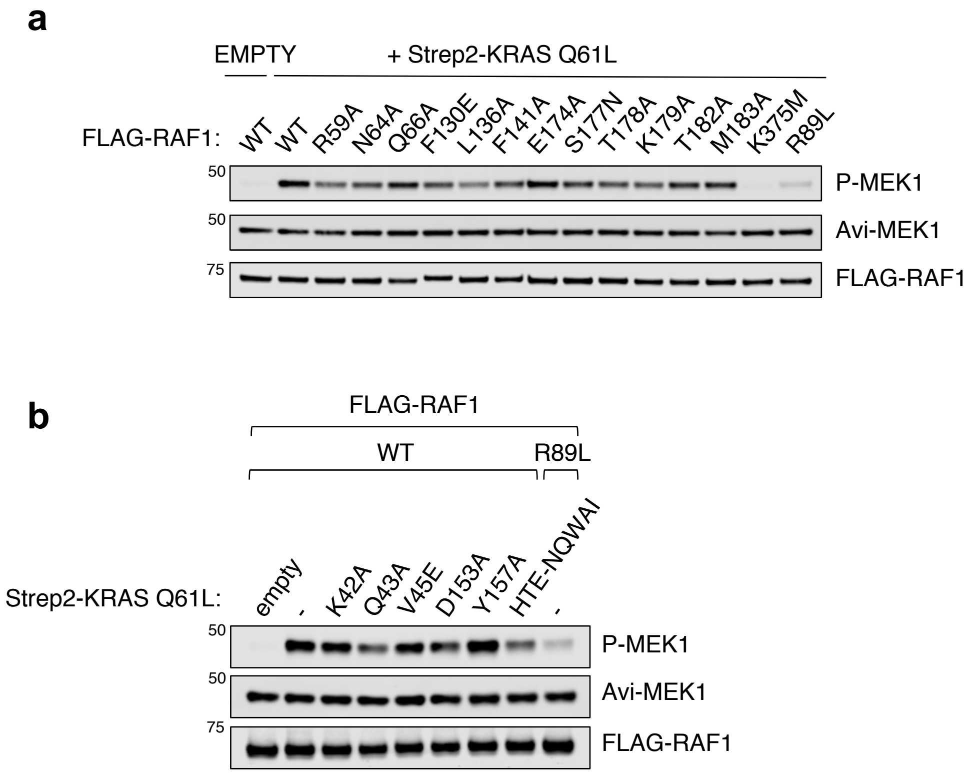

Supplementary Figure 7: Interactions at the RAF1-CRD interface with KRAS required for full RAF kinase activity. **(a)** Wild-type and mutants of RAF1 (purified from FLAG IPs in Figure 5a) were used to monitor kinase activity towards recombinant MEK1. **(b)** Kinase activity of RAF1 (from Figure 5c) was measured was measured in response to KRAS mutants in the same manner as before. KRAS mutations were introduced into the constitutively active KRAS-Q61L background and "-" denotes Q61L with no additional mutations. Representative of n = 3 experiments.

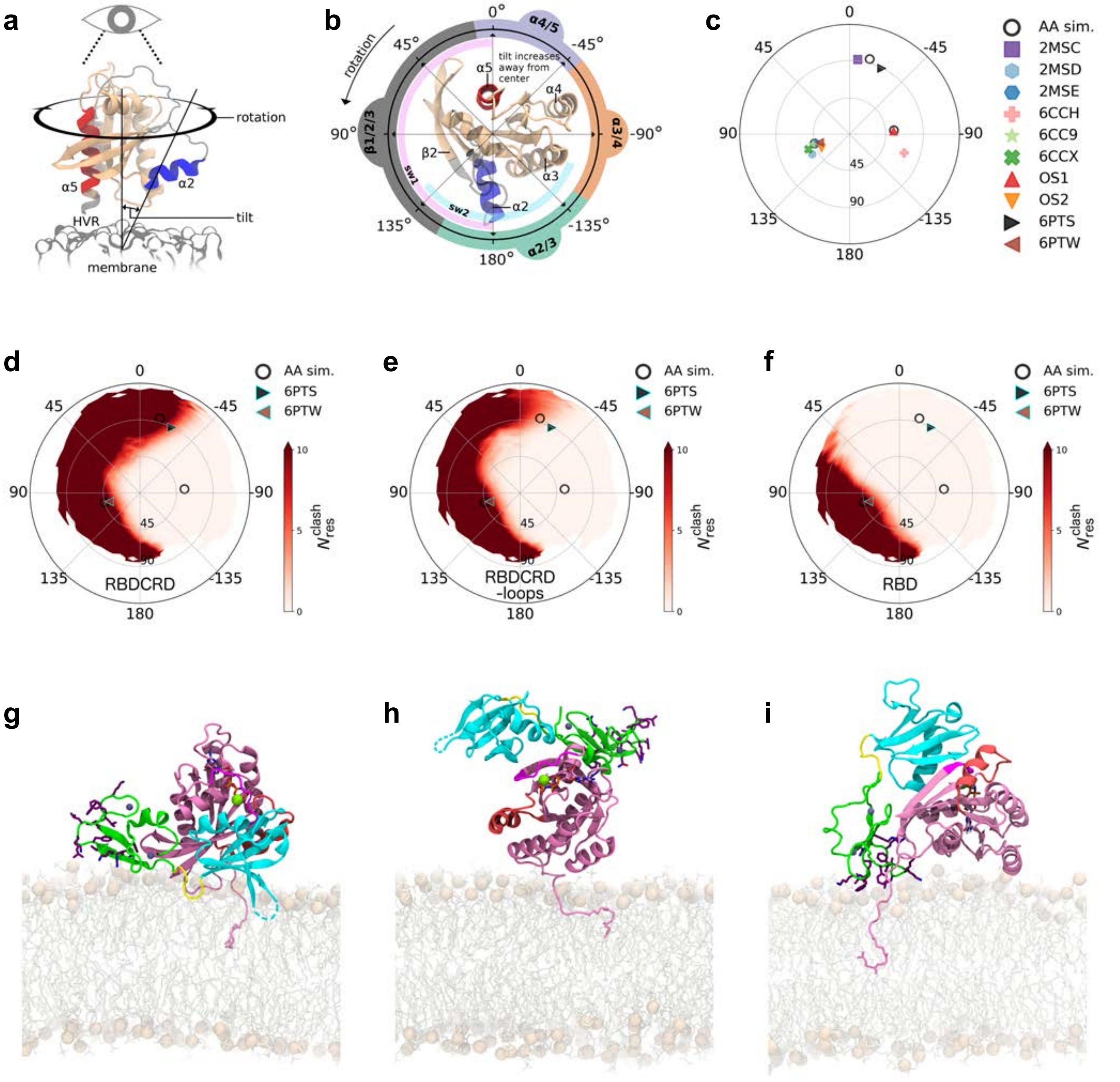

Supplementary Figure 8: Orientation of KRAS in relation to the membrane surface and occlusion of RAF1 binding. (**a, b**) Definition of tilt and rotation angles. Regions of KRAS that approach the membrane upon tilting are inscribed around the perimeter of panel b. Reprinted with permission from *Biophysical Journal*[1]. (**c**) Reported membrane orientations of KRAS. "AA sim." are from simulations of KRAS on mixed anionic: zwitterionic lipid bilayers[1]. PDB: 2MSC, 2MSD, and 2MSE are the ''exposed,'' ''occluded,'' and ''semi-occluded'' orientations from Mazhab-Jafari *et al.*[2]; PDB: 6CCH, 6CC9, and 6CCX are the ''E3,'' ''O1,'' and ''O2'' orientations from Fang *et al.*[3] ''OS1'' and ''OS2'' are from simulations by Prakash *et al.*[4]. PDB: 6PTS and 6PTW are the "state A" and "state B" orientations of KRAS-RBDCRD from Fang *et al.*[5]. Ensemble average orientations are reported for PDB entries comprising multiple models. (**d-f**) Relationship between KRAS orientation and steric competence to bind (d) RBDCRD, (e) RBDCRD excluding the CRD's mixed hydrophobic/cationic loop residues ^{143}RKTFLKLAF^{151} and ^{157}KFLLNGFR^{164}, and (f) RBD. $N_{\mathrm{res}}^{\mathrm{clash}}$ is the

number of RAF1 residues that overlap with membrane lipids (any protein atom within 0.1 nm of any lipid atom). Relatively large values of, shown as red in panels d-f, indicate occlusion of protein-protein interactions by approximately planar models of the cell membrane. In some cases, this occlusion may be relieved by structural accommodation of membrane lipids. The difference between panels e and f represents evaluation of occlusion according to models in which hydrophobic CRD loops are (e) disallowed or (f) allowed to penetrate the membrane surface. (**g-h**) Additional models of membrane interactions of the crystallographic RAS-RBDCRD complex, analogous to Fig. 7c except orienting KRAS such that (g) β sheets β1-3 and switch-I or (h) α helices α3 and α4 face the membrane. (**i)** Similar to Fig. 7c, except KRAS and RAF1 are oriented and configured as in model 0 of PDB: 6PTS[5]. Source data for panels c, d, e, and f are provided as a Source Data file.

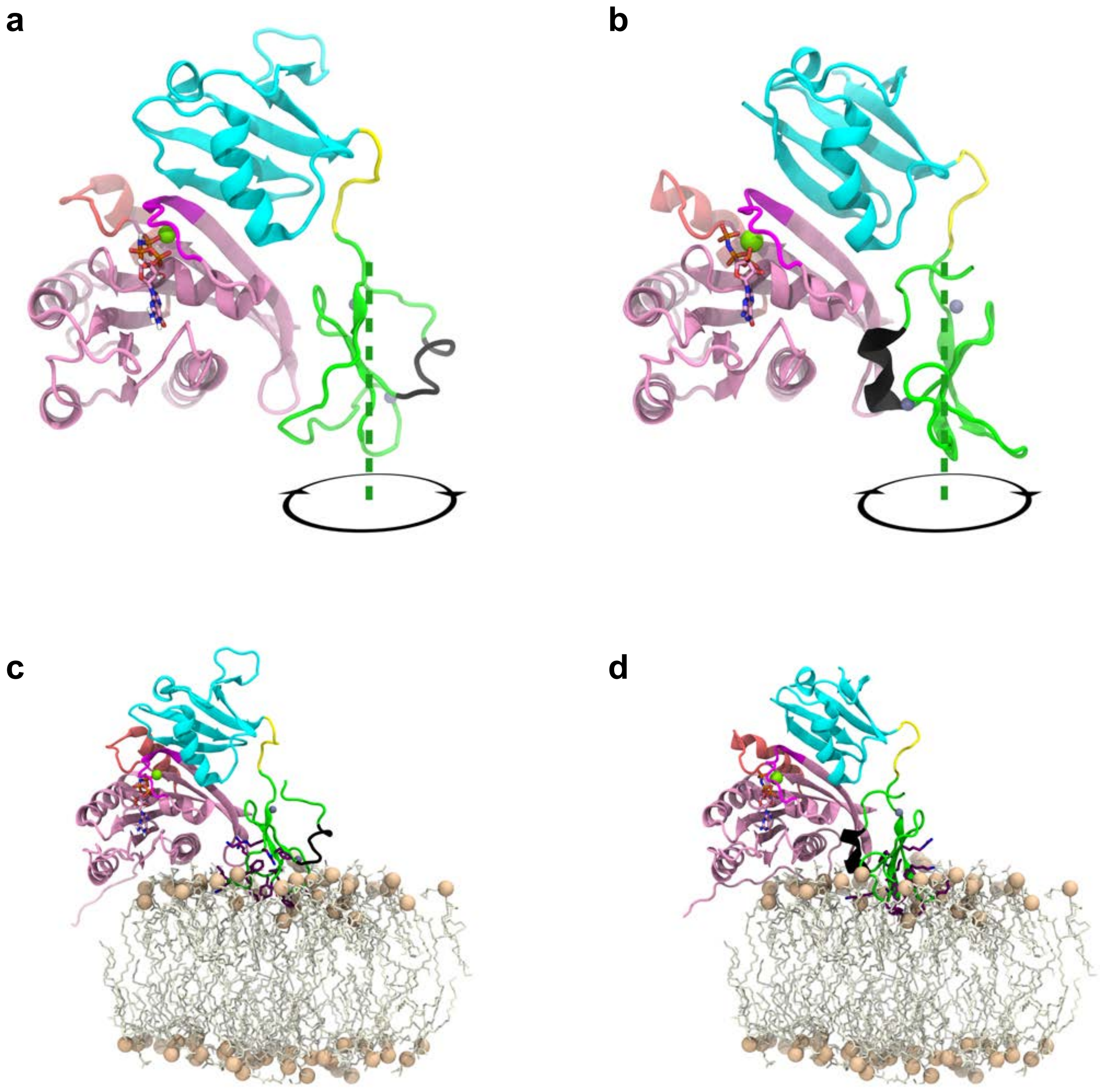

Supplementary Figure 9: Influence of the KRAS-CRD interface on CRD-membrane contacts. **(a, b)** Structural comparison of KRAS-RBDCRD in (a) NMR data-driven model from Fang *et al.* [5] and (b) crystal structure of KRAS-RBDCRD presented in this work. Note the ~180° rotation of the CRD about its long axis in panel a vs panel b. Helical CRD residues ^{174}EHCSTKV^{180} are shown in black. **(c)** PDB: 6PTS model 0 from Fang *et al.*[5]. **(d)** Crystallographic KRAS-RBDCRD aligned on KRAS G-domain C_α atoms from PDB: 6PTS model 0, showing lipids from the later model.

Supplementary Table 1: Crystallization conditions for the structures described in this study.

**KRAS-RAF1 structures**	**Crystallization condition**
KRAS-RAF1(RBD)	0.09 M Halogens, 0.1M Imidazole.MES pH 6.5, 37.50% MPD, PEG 1000, PEG3350
KRAS-RAF1(RBDCRD). Crystal form I	2.2 M $AMSO_4$, 6.5% (w/v) PEG 400, pH 5.3
KRAS-RAF1(RBDCRD) Crystal form II	100 mM sodium cacodylate pH 6.5, 200 mM sodium citrate, 15% 2-propanol, 0.25% (w/v) n-octyl-beta-D-glucoside, 0.35 mM D-myo-phosphatidylinositol 3,4,5-triphosphate, and 0.25% (w/v) n-dodecyl-beta-D-maltoside
$KRAS^{G12V}$-RAF1(RBDCRD)	100 mM sodium cacodylate pH 6.5, 200 mM $MgCl_2$, 8% PGA, 0.35 mM D-myo-phosphatidylinositol 3,4,5-triphosphate
$KRAS^{G13D}$-RAF1(RBDCRD)	100 mM sodium cacodylate pH 6.5, 700 mM sodium acetate, 0.35 mM D-myo-phosphatidylinositol 3,4,5-triphosphate
$KRAS^{Q61R}$-RAF1(RBDCRD)	100 mM sodium cacodylate pH 6.5, 200 mM $MgCl_2$, 8% PGA-LM

Supplementary Table 2: List of protein-protein interactions present at the KRAS-RAF1(RBD) interface in the structure of KRAS-RAF1(RBDCRD) complex.

S. No:	KRAS	atom	RBD	atom	Distance
**H-bonds**					
1	D33	OD2	K84	NZ	3.13
2	E37	O	V69	N	2.97
3	E37	OE2	R67	NE	2.95
4	D38	OD1	T68	OG1	2.65
5	D38	OD1	R89	NH2	2.93
6	S39	N, OG	R67	O, N	2.86 – 3.03
7	S39	O	R89	NE, NH2	2.92 – 3.13
8	R41	N	Q66	OE1	3.10
9	R41	NH2	N64	OD1	3.04
**Salt-bridge**					
1	E31	OE2	K84	NZ	3.37
2	D33	OD2	K84	NZ	3.13
3	E37	OE2	R67	NE	2.95
4	D38	OD1	R89	NH2	2.93
**Non-bonded interactions**					
1	I24	CG2	V88	O	3.52
2	Q25	CB, CG, CD, OE1	V88	O, CA, CG1	3.6 – 3.81
3	Q25	NE2	K87	C, CD, NZ	3.38 – 3.79
4	V29	CG1	K84	NZ	3.55
5	E31	CG, CD, OE2	K84	NZ, CE	3.37 – 3.87
6	D33	CB, CG, OD2	K84	NZ, CD	3.13 – 3.84
7	I36	CG2	V69	CB, CG1	3.64 – 3.73
8	I36	CD1	T57	CG2	3.54
9	E37	O	T68	CA, C, OG1	3.32 – 3.76
10	E37	O, CB	V69	N, CG2	2.97 – 3.82
11	E37	CD, OE2	R67	NE, CD, CG	2.95 – 3.82
12	E37	OE2	R59	CD	3.79
13	D38	CA, C	R67	O	3.45 – 3.61
14	D38	CG, OD1, OD2	T68	OG1, CA, CB	2.65 – 3.71
15	D38	CB, CG, OD1	R89	NH1, NH2, CZ	2.83 – 3.83
16	S39	N, CA, O, OG, CB	R67	O, N, CG, NH1	2.86 – 3.89
17	S39	N, C, O	R89	NH2, NE, CZ	2.92 – 3.82
18	S39	O, OG	Q66	CB, CG, OE1, CA, C	3.32 – 3.90
19	Y40	CA, C	Q66	OE1	3.25 – 3.63
20	Y40	CD2, CE2, CZ	R89	CG, NE, CZ, NH2, NH1	3.45 - 3.85
21	Y40	CE2	V88	CG2	3.61
22	R41	N, CG	Q66	CD, OE1, CG	3.1 – 3.86
23	R41	CB, CG, CD, NE, CZ, NH2	N64	ND2, O, OD1	3.04 - 3.88
24	R41	NH2	K65	OD1	3.86
25	L56	CD1	R67	NH2	3.79

Supplementary Table 3: List of protein-protein interactions present at the KRAS-RAF1(RBD) interface in the structure of KRAS-RAF1(RBD) complex.

S. No:	KRAS	atom	RBD	atom	Distance
**H-bonds**					
1	E31	OD2	K84	NZ	2.77
2	E37	O	V69	N	3.11
3	E37	OE1, OE2	R67	NE, NH2	2.74 - 3.07
4	E37	OE2	R59	NE, NH2	2.64 - 2.82
5	D38	OD1	T68	OG1	2.61
6	D38	OD1	R89	NH2	2.91
7	S39	N, OG	R67	O, N	2.91 - 2.94
8	S39	O	R89	NE, NH2	2.89 - 3.07
9	R41	N	Q66	OE1	3.11
10	R41	NH1	N64	OD1	2.95
**Salt-bridge**					
1	E31	OE2	K84	NZ	2.77
2	E37	OE2	R59	NE	2.64
3	E37	OE1	R67	NE	2.74
4	D38	OD1	R89	NH2	2.91
**Non-bonded interactions**					
1	I24	CG2	V88	O	3.31
2	Q25	OE1	V88	CG1	3.71
3	E31	CG, CD, OE2	K84	NZ, CE	2.77 - 3.55
4	I36	CB, CD1	V69	CG1	3.89 - 3.9
5	I36	CD1	T57	CG2	3.89
6	E37	O	T68	CA, C	3.42 - 3.63
7	E37	O, CG, OE2	V69	N, CG2	3.11 - 3.81
8	E37	CD, OE2	R59	NE, NH2, CD, CZ	2.64 - 3.84
9	E37	CD, OE1, OE2	R67	NE, NH2, CB, CD, CZ	2.74 - 3.88
10	D38	CA, C, OD1	R67	O	3.33 - 3.84
11	D38	CG, OD1, OD2	T68	OG1, CA, CB, OG1	2.61 - 3.67
12	D38	CG, OD1	R89	NH1, CZ, NH2	2.91 - 3.86
13	S39	N, O, OG	R67	O, N, CA, CB	2.91 - 3.83
14	S39	N, C, O	R89	NH2, NE, CZ	2.89 - 3.78
15	S39	O, OG	Q66	CB, CG, CA, C	3.41 - 3.6
16	Y40	CA, C	Q66	OE1	3.31 - 3.64
17	Y40	CD2, CE2, CZ, OH	R89	CG, NE, CZ, NH2, NH1	3.37 - 3.84
18	Y40	CE2	V88	CG2	3.72
19	R41	N	Q66	CD, OE1	3.11 - 3.8
20	R41	CB, CG, CD, NH1	N64	ND2, O, CG, OD1	2.95 - 3.84
21	R41	CZ, NH1, NH2	K65	CD	3.53 - 3.77
22	M67	SD	R59	NH2	3.89

Supplementary Table 4: List of interdomain interactions present at the RBD-Linker-CRD interface in the structure of KRAS-RAF1(RBDCRD) complex.

S. No:	RBD	atom	CRD	atom	Distance
**H-bonds (Not observed)**					
**Salt-bridge (Not observed)**					
**Non-bonded interactions**					
1	G90	O	M183	CG	3.40
2	L91	CD2	M183	SD	3.84

S. No:	RBD	atom	Linker	atom	Distance
**H-bonds**					
1	L131	O	V134	C	2.77
**Salt-bridge (Not observed)**					
**Non-bonded interactions**					
1	F130	CB	P135	CA	3.69
2	F130	CG, CD1, CE1, CE2, CZ	L136	N, CB, O	3.38 - 3.77
3	L131	C, O, CB	V134	N, CA, C, O, CB, CG2	3.35 - 3.89
4	L131	O	P135	N	3.86

S. No:	Linker	atom	CRD	atom	Distance
**H-bonds**					
1	T137	O	C184	N	3.19
2	T137	OG1	T138	N, O	2.67 - 3.19
**Salt-bridge (Not observed)**					
**Non-bonded interactions**					
1	T137	O, CG2	C184	N, CA, O, CB	3.19 - 3.90
2	T137	C, CB, OG1, CG2	T138	N, CA, C, O, CB, OG1	2.67 - 3.87

Supplementary Table 5: List of protein-protein interactions present at the KRAS-RAF1(CRD) interface in the structure of KRAS-RAF1(RBDCRD) complex.

S. No:	KRAS	atom	CRD	atom	Distance
**H-bonds**					
1	K42	NZ	T178	O	3.23
2	K42	NZ	V180	O	2.68
3	Q43	NE2	H139	O	2.92
4	V45	N	S177	OG	3.15
5	D47	N	E174	OE2	2.77
6	G48	N	E174	OE1	2.71
7	G48	O	R143	NH1	3.11
8	R149	NH2	T178	OG1	2.99
9	D153	OD1	T178	OG1	2.81
**Salt-bridge (Not observed)**					
**Non-bonded interactions**					
1	L23	O, CD1	T178	O, CG2	3.18 - 3.73
2	I24	O	T182	OG1	3.44
3	N26	CG, OD1, ND2	K179	CG, CD, CE	3.72 – 3.88
4	R41	O	T182	CG2	3.74
5	K42	CD, NZ	T182	OG1	3.63 - 3.73
6	K42	CE, NZ	T178	O	3.23 - 3.49
7	K42	CE, NZ	V180	O, C	2.68 – 3.86
8	Q43	CD, OE1, NE2	H139	O, C, N	2.92 – 3.80
9	Q43	NE2	T138	CB	3.66
10	Q43	OE1	F141	CD2	3.91
11	V44	CG1	S177	OG	3.75
12	V44	CG1	T178	CG2	3.79
13	V45	N, CA, O	S177	CB, OG	3.15 – 3.90
14	V45	O	E174	CG, O, CD	3.36 – 3.79
15	V45	CG1	F163	CE1, CZ	3.42 – 3.48
16	I46	C, CA	E174	OE2	3.71 – 3.80
17	D47	N, CA, C	E174	CD, OE1, OE2	2.77 - 3.89
18	G48	N, CA	E174	CD, OE1	2.71 - 3.59
19	G48	CA, C, O	R143	NH1	3.11 – 3.85
20	G48	O	F163	CZ	3.74
21	R149	CZ, NH1, NH2	T178	OG1, CB	2.99 – 3.75
22	R149	NH1	K179	CE	3.76
23	D153	CG, OD1, OD2	T178	OG1, CG2, CB	2.81 - 3.90
24	D153	OD2	H175	O	3.77
25	Y157	CE1	T178	CG2	3.65
26	Y157	OH	E174	O	3.27
27	Y157	CE2	H175	CD2, NE2	3.86 – 8.88
28	Y157	OH	S177	OG	3.47

SUPPLEMENTARY REFERECES

1. Neale, C. & Garcia, A.E. The Plasma Membrane as a Competitive Inhibitor and Positive Allosteric Modulator of KRas4B Signaling. *Biophys J* **118**, 1129-1141 (2020).

2. Mazhab-Jafari, M.T. et al. Oncogenic and RASopathy-associated K-RAS mutations relieve membrane-dependent occlusion of the effector-binding site. *Proc Natl Acad Sci U S A* **112**, 6625-30 (2015).

3. Fang, Z. et al. Inhibition of K-RAS4B by a Unique Mechanism of Action: Stabilizing Membrane-Dependent Occlusion of the Effector-Binding Site. *Cell Chem Biol* **25**, 1327-1336 e4 (2018).

4. Prakash, P., Zhou, Y., Liang, H., Hancock, J.F. & Gorfe, A.A. Oncogenic K-Ras Binds to an Anionic Membrane in Two Distinct Orientations: A Molecular Dynamics Analysis. *Biophys J* **110**, 1125-38 (2016).

5. Fang, Z. et al. Multivalent assembly of KRAS with the RAS-binding and cysteine-rich domains of CRAF on the membrane. *Proc Natl Acad Sci U S A* **117**, 12101-12108 (2020).
