# Supplementary material for: KRAS interaction with RAF1 RAS-binding domain and cysteine-rich domain provides insights into RAS-mediated RAF activation
Source: Nat Commun. 2021 Feb 19;12:1176. doi: 10.1038/s41467-021-21422-x (PMC7895934; doi:10.1038/s41467-021-21422-x)
Supplement: Supplementary file 3 — Reporting Summary [file 41467_2021_21422_MOESM3_ESM.pdf]

nature research

Corresponding author(s): Dhirendra Simanshu, Frank McComick

Last updated by author(s): 01/21/2021

Reporting Summary

Nature Research wishes to improve the reproducibility of the work that we publish. This form provides structure for consistency and transparency in reporting. For further information on Nature Research policies, see our Editorial Policies and the Editorial Policy Checklist.

Statistics

For all statistical analyses, confirm that the following items are present in the figure legend, table legend, main text, or Methods section.

n/a	Confirmed	
☐	☒	The exact sample size (*n*) for each experimental group/condition, given as a discrete number and unit of measurement
☒	☐	A statement on whether measurements were taken from distinct samples or whether the same sample was measured repeatedly
☒	☐	The statistical test(s) used AND whether they are one- or two-sided<br>*Only common tests should be described solely by name; describe more complex techniques in the Methods section.*
☒	☐	A description of all covariates tested
☒	☐	A description of any assumptions or corrections, such as tests of normality and adjustment for multiple comparisons
☐	☒	A full description of the statistical parameters including central tendency (e.g. means) or other basic estimates (e.g. regression coefficient) AND variation (e.g. standard deviation) or associated estimates of uncertainty (e.g. confidence intervals)
☒	☐	For null hypothesis testing, the test statistic (e.g. *F*, *t*, *r*) with confidence intervals, effect sizes, degrees of freedom and *P* value noted<br>*Give P values as exact values whenever suitable.*
☒	☐	For Bayesian analysis, information on the choice of priors and Markov chain Monte Carlo settings
☒	☐	For hierarchical and complex designs, identification of the appropriate level for tests and full reporting of outcomes
☒	☐	Estimates of effect sizes (e.g. Cohen's *d*, Pearson's *r*), indicating how they were calculated

Our web collection on statistics for biologists contains articles on many of the points above.

Software and code

Policy information about availability of computer code

Data collection	Crystallographic data sets were collected at 24-ID-C/E beamlines at the Advanced Photon Source (APS), Argonne National Laboratory, and integrated and scaled using the program XDS (version 20200417). Structure solution, model building and refinement were carried out using programs Phaser (version 2.7.17), COOT (version 0.9) and Phenix.Refine (version 1.17.1_3660), respectively.
Data analysis	All of the structural figures were rendered in PyMOL 2.4.0 (Schrödinger, LLC) or VMD (version 1.9.4) with secondary structural elements assigned using the DSSP server (http://swift.cmbi.ru.nl/gv/dssp/). The amino acid sequence alignments were carried out using Clustal Omega, and the figures were produced using ESPript 3.0. Quantification of band intensity in Western blot was performed with ImageStudio Lite (Li-COR) software (version 5.0). SPR sensorgram data was analyzed using using the Biacore Evaluation software (version 2.x).

For manuscripts utilizing custom algorithms or software that are central to the research but not yet described in published literature, software must be made available to editors and reviewers. We strongly encourage code deposition in a community repository (e.g. GitHub). See the Nature Research guidelines for submitting code & software for further information.

Data

Policy information about availability of data

All manuscripts must include a data availability statement. This statement should provide the following information, where applicable:

- Accession codes, unique identifiers, or web links for publicly available datasets
- A list of figures that have associated raw data
- A description of any restrictions on data availability

The atomic coordinates and structure factors of the various complexes have been deposited in the Protein Data Bank and are available under accession numbers: 6XI7 – KRAS in complex with RAF1(RBDCRD), crystal form I; 6XHB – KRAS in complex with RAF1(RBDCRD), crystal form II; 6VJJ - KRAS in complex with RAF1(RBD); 6XHA – KRAS-G12V in complex with RAF1(RBDCRD); 6XGV – KRAS-G13D in complex with RAF1(RBDCRD), and 6XGU- KRAS-Q61R in complex with RAF1(RBDCRD). For

the structural comparison and analysis described in this study, we used publicly available structures with PDB ID 4G0N and 1FAR present in the Protein Data Bank. All data needed to evaluate the conclusions in the paper are present in the paper and/or the Supplementary Information. Additional data related to this paper may be requested from the authors. Source data are provided with this paper.

Field-specific reporting

Please select the one below that is the best fit for your research. If you are not sure, read the appropriate sections before making your selection.

[x] Life sciences [ ] Behavioural & social sciences [ ] Ecological, evolutionary & environmental sciences

For a reference copy of the document with all sections, see nature.com/documents/nr-reporting-summary-flat.pdf

Life sciences study design

All studies must disclose on these points even when the disclosure is negative.

Sample size	Biochemical and cellular data shown were from at least three independent experiments. The sample size was sufficient to represent reproducibility and credibility in the biochemical and cellular assays.
Data exclusions	No data were excluded from analysis.
Replication	Experiments involving mammalian cells were performed a minimum of 3 times and all results were reproducible. Numbers of replications in different experiments can be found in the corresponding figure legends and source Data file.
Randomization	Not applicable to the experimental design because no grouping was needed.
Blinding	Not applicable to the experimental design as no grouping was needed for this study.

Reporting for specific materials, systems and methods

We require information from authors about some types of materials, experimental systems and methods used in many studies. Here, indicate whether each material, system or method listed is relevant to your study. If you are not sure if a list item applies to your research, read the appropriate section before selecting a response.

Materials & experimental systems

n/a	Involved in the study	
[ ]	[x]	Antibodies
[ ]	[x]	Eukaryotic cell lines
[x]	[ ]	Palaeontology and archaeology
[x]	[ ]	Animals and other organisms
[x]	[ ]	Human research participants
[x]	[ ]	Clinical data
[x]	[ ]	Dual use research of concern

Methods

n/a	Involved in the study	
[x]	[ ]	ChIP-seq
[x]	[ ]	Flow cytometry
[x]	[ ]	MRI-based neuroimaging

Antibodies

Antibodies used	Avi-tag - GenScript A01738 (1:5000); MEK1 - SCBT sc-6250 (1:500); FLAG - Sigma F7425 (1:4000); MEK P-S217/221 – Cell Signaling 9121 (1:2000); RAF1 - BD Transduction Laboratories 610152 (1:2000); NWSHPQFEK (Strep-tag) -GenScript A00626 (1:5000).
Validation	Avi Tag Antibody recognizes C-terminal Avi tagged fusion proteins and slightly recognizes N-terminal Avi tagged fusion proteins in Western Blot. MEK1 Antibody is recommended for detection of MEK1 by Western Blot, immunoprecipitation, immunofluorescence, immunohistochemistry and ELISA. Species: mouse, rat, human, bovine, and others. FLAG Antibody recognizes the FLAG epitope located on FLAG fusion proteins (N-terminal, N-terminal-Met, and C-terminal FLAG fusion proteins) by Western Blot, immunoprecipitation, and immunofluorescence. Phospho-MEK1/2 (Ser217/221) Antibody detects endogenous levels of MEK1/2 only when activated by phosphorylation at Ser217/221. This antibody does not cross-react with related kinases including activated SEK (MKK4), MKK3 or MKK6. It will also react with MEK1/2 singly phosphorylated at Ser217 and singly phosphorylated at Ser221. For use in Western Blot and immunoprecipitation. Species: human, mouse, rat, monkey, S. cerevisiae. Raf1 recognizes endogenous Raf1 by Western blot, immunoprecipitation or immunofluorescence. Species: human, mouse, rat, dog, chicken. NWSHPQFEK recognizes NWSHPQFEK (Strep2) tagged fusion protein by Western blot and ELISA.

Eukaryotic cell lines

Policy information about cell lines

Cell line source(s)	HEK-293T cells were from ATCC

Authentication	No cell authentication method was used.
Mycoplasma contamination	All cell lines used tested negative for mycoplasma.
Commonly misidentified lines (See ICLAC register)	No misidentified lines were used.
